# Effect of postoperative high load long duration inspiratory muscle training on pulmonary function and functional capacity after mitral valve replacement surgery: A randomized controlled trial with follow-up

**DOI:** 10.1371/journal.pone.0256609

**Published:** 2021-08-27

**Authors:** Fatma A. Hegazy, Sara M. Mohamed Kamel, Ahmed S. Abdelhamid, Emad A. Aboelnasr, Mahmoud Elshazly, Ali M. Hassan

**Affiliations:** 1 Department of Physiotherapy, Collage of Health Sciences, University of Sharjah, Sharjah, UAE; 2 Faculty of Physical Therapy, Cairo University, Giza, Egypt; 3 Department of Physical Therapy, National Heart Institute, Cairo, Egypt; 4 Department of Physical Therapy for Musculoskeletal Disorders and Its Surgeries, Faculty of Physical Therapy, South Valley University, Qena, Egypt; 5 Department of Physical Therapy for Surgery, Faculty of Physical Therapy, South Valley University, Qena, Egypt; 6 Department of Physical Therapy for Internal Medicine and Geriatrics, Faculty of Physical Therapy, South Valley University, Qena, Egypt; Prince Sattam Bin Abdulaziz University, College of Applied Medical Sciences, SAUDI ARABIA

## Abstract

**Objectives:**

Although, pre-operative inspiratory muscle training has been investigated and reported to be an effective strategy to reduce postoperative pulmonary complications, the efficacy of postoperative inspiratory muscle training as well as the proper load, frequency, and duration necessary to reduce the postoperative pulmonary complications has not been fully investigated. This study was designed to investigate the effect of postoperative high-load long-duration inspiratory muscle training on pulmonary function, inspiratory muscle strength, and functional capacity after mitral valve replacement surgeries.

**Design:**

Prospective randomized controlled trial.

**Methods:**

A total of one hundred patients (mean age 38.3±3.29years) underwent mitral valve replacement surgery were randomized into experimental (n = 50) and control (n = 50) groups. The control group received conventional physiotherapy care, while experimental group received conventional care in addition to inspiratory muscle training, with 40% of the baseline maximal inspiratory pressure targeting a load of 80% by the end of the 8 weeks intervention protocol. Inspiratory muscle training started on the patient’s first day in the inpatient ward. Lung functions, inspiratory muscle strength, and functional capacity were evaluated using a computer-based spirometry system, maximal inspiratory pressure measurement and 6MWT respectively at 5 time points and a follow-up assessment was performed 6 months after surgery. Repeated measure ANOVA and post-hoc analyses were used (p <0.05).

**Results:**

Group-time interactions were detected for all the studied variables (p<0.001). Between-group analysis revealed statistically significant postoperative improvements in all studied variables in the experimental group compared to the control group (p <0.001) with large effect size of η^2^ ˃0.14. Within-group analysis indicated substantial improvements in lung function, inspiratory pressure and functional capacity in the experimental group (p <0.05) over time, and these improvements were maintained at follow-up.

**Conclusion:**

High intensity, long-duration postoperative inspiratory muscle training is highly effective in improving lung function, inspiratory muscle strength, and functional capacity after mitral valve replacement surgeries.

## Introduction

There is a high incidence of postoperative pulmonary complications (PPC) occurring after Mitral valve replacement surgery due to many factors such as general anesthesia, median sternotomy, cardiopulmonary bypass machine and thoracic manipulation [1]. These complications include atelectasis, pleural effusion, pneumothorax, bronchospasm, lung secretions, respiratory infection, pneumonia, respiratory failure, pulmonary embolism and acute respiratory distress syndrome.

Furthermore, cardiac surgeries severely affect respiratory mechanics, decreases lung volumes, affect gas exchange, contribute to changes in the ventilation/perfusion ratio reduce the cardiorespiratory capacity and induce physical inactivity, hence leading to a reduction in functional capacity [2–5].

Cardiopulmonary physical therapy techniques to prevent [PPC] can be categorized into those delivered preoperatively, postoperatively, or both. Interventions include early mobilization, breathing exercises, coughing techniques, incentive spirometry, and respiratory muscle training. Inspiratory muscle training (IMT) has shown potential beneficial effects in cardiac patients undergoing coronary artery bypass surgery [2, 3].

Several studies have been conducted to elucidate the potential effectiveness of IMT as well as its preventive effects, on developing PPC, however, most of these studies analyzed pre-operative IMT only [3, 6]. In their systematic review, Kendall et al. [7], reported that there is a paucity of the available literature investigating the effectiveness of postoperative IMT and its proper dose prescription [ie, starting load, maximal achieved load, load increment, intervention duration, frequency, duration of sessions and the degree of supervision]. Thus, it is difficult to ascertain the effectiveness and the preventive efficacy of IMT in reducing the risk of developing PPC.

Up to the authors’ knowledge, few studies have investigated the effectiveness of postoperative IMT and these studies have differed in terms of methodology, sample size, protocols and durations of intervention with some supporting one week of intervention [8], two weeks of interventions [6] while others investigated the effect of four weeks of intervention [9]. Different methodologies and unclear IMT parameters have made it difficult to compare between previous studies and to make clinical recommendations for administrating postoperative IMT.

It has been reported that high-load pre-operative IMT is related to reduction in PPC [3, 7] however, up to the authors’ knowledge, there is no previous study has investigated the effectiveness of a range of postoperative IMT doses. Since the benefits of IMT are likely to be related to the prescribed dose postoperatively [7], this study was designed to investigate the effects of high-load long-duration (8 week) supervised postoperative IMT on pulmonary function, inspiratory muscle strength, and functional capacity after mitral valve replacement. We hypothesized that high-load postoperative IMT would effectively preserve pulmonary function, inspiratory muscle strength, and functional capacity after mitral valve replacement surgeries.

## Methods

### Study design

This study was conducted as a parallel-group, single-blind, single-center, randomized controlled trial with a follow-up of postoperative outcomes.

### Participants

All participants were patients aged 25–50 years recruited from the waiting list for elective Mitral valve replacement (MVRS) at South Valley university hospital, Egypt. The current study had been conducted between June 2019 and January 2020.

The inclusion criteria included 1) patients undergoing elective MVRS, due to rheumatic heart disease with moderate [1.0–1.4 cm^2^] to severe [less than 1.0 cm^2^] stenosis, calcified mitral valve leaflet or significant mitral valve regurgitation; 2) aged 25–50 years; 3) normal or over weight with body mass index (BMI) rang 18.5–29.9 kg/m^2^; 4) post-operative medically and clinically stable; 5) able to walk independently without any assistive devices. The diagnosis had been performed according to the guideline of the American College of Cardiology and American Heart Association for the management of patients with valvular heart disease [10]. The participants were diagnosed by an experienced cardiologist with more than 12 years of clinical experience.

The exclusion criteria were; 1) obesity (BMI ˃ 30kg/m^2^); 2) Patients with any other significant valvar or coronary artery diseases necessitating surgical intervention 3) postoperative hemodynamic complications such as lung congestion or myocardial infarction; 4) history of pulmonary diseases or infection such as chronic obstructive pulmonary diseases (COPD) or tuberculosis; 5) smokers; 6) post-operative renal or hepatic failure; 7) Cardiac arrhythmia or unstable angina; 8) uncontrolled hypertension; 9) post-operative hemodynamic instability; 10) post-operative prolonged ventilation [more than 24 hours]; 11) neurological/ musculoskeletal disorders that could affect the functional capacity or pulmonary function such as(parkinsonism, ankylosing spondylitis, scoliosis, ….etc); 12) history of previous cardiothoracic surgery.

### Ethics

The current study had been carried out in accordance with The Code of Ethics of the World Medical Association (Declaration of Helsinki), and was approved by the Human Research Ethics Committee of South Valley University. This study had been registered in the Pan African Clinical Trial Registry with the following number: PACTR202006495227440. The trial was registered after starting participants’ recruitment due to COVID-19 pandemic circumstances. The authors confirm that all ongoing and related trials for this intervention are registered. The current study has been reported in line with Consolidated Standards of Reporting Trials (CONSORT) statement guidelines for reporting randomized trials (S1 Checklist). The study protocol is presented as a supporting file (S1 File).

Written informed consents were obtained from participants before enrollment in the study. All participants were informed about the study objectives before participation. Participants were informed about their rights to refrain from the study at any time if they want.

### Randomization, allocation concealment and blinding

The patients were randomly selected from the list of patients who were scheduled for elective MVRS using simple randomization method. After surgery, random allocation of patients was performed through sequentially numbered sealed opaque envelopes.

**Fig 1:**
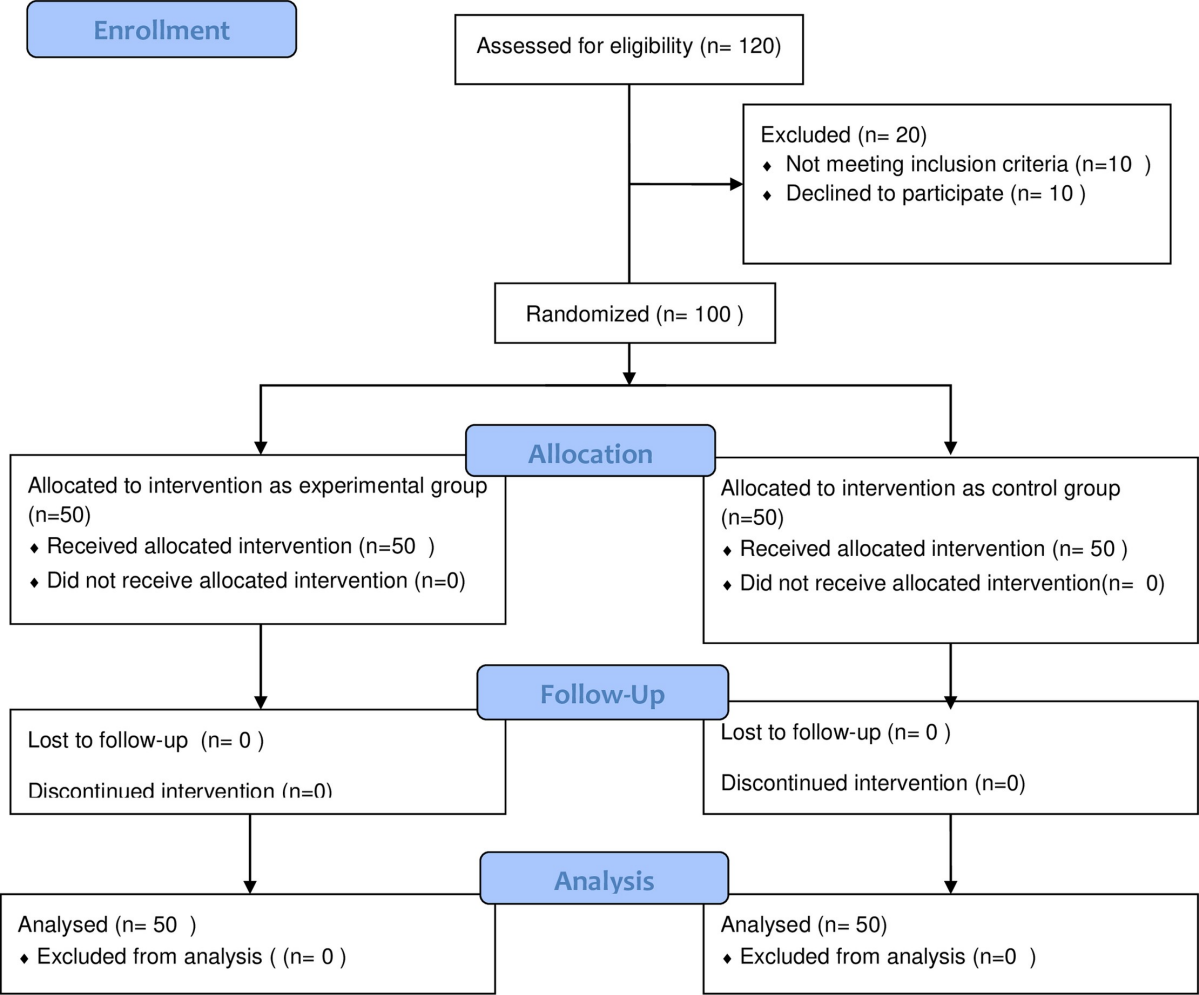
Enrollment flow chart.

The sequentially numbered envelopes were arranged and sealed by an independent statistician who did not involve in the study and was blinded to the study objectives. These envelopes were given to the allocator who was a physiotherapy practitioner, and he was blinded to the study objectives and did not participate either in the intervention or the assessment procedures. The allocation ratio was 1:1. Each envelope was opened only in front of the patient to be assigned. The patients were blinded to the group allocation. Fifty participants were randomly allocated to each of the experimental group and control group. The study Recruitment process of the participants was shown in the enrollment flow chart (Fig 1). The demographic characteristics of the study participants were presented in Table 1.

**Table 1:** Baseline demographic, clinical and operative characteristics of the participants in both study and control groups.

Variables	Control Group(n = 50)	Study Group(n = 50)	P Value
**Age (years)**	38.3 (3.29)	39 (4.28)	0.45^a^
**Gender**			
**Male**	26 (52%)	25 (50%)	0.56^b^
**Female**	24 (48%)	25 (50%)	0.42^b^
**BMI (Kg/m** ^**2**^ **)**	28.23 (1.89)	27.32(1.4)	0.84^a^
**Atrial Fibrillation**	34(68%)	36(72%)	0.74^b^
**Sinus rhythm**	16(32%)	14(28%)	0.65^b^
**NYHA Functional classification**			
**Class II**	17(34%)	19(32%)	0.24^b^
**Class III**	25(50%)	23(46%)	0.51^b^
**Class IV**	8(16%)	8(16%)	0.34^b^
**Endocardiographic characteristics**			
**LVEDD (cm)**	6.45(0.51)	6.40(0.48)	0.75^a^
**LVESD (cm)**	3.88(0.39)	4.03(0.56)	0.66^a^
**PASP (mmHg)**	64.77(14.43)	63.65(10.77)	0.78^a^
**Mitral Valve orifice area (cm** ^**2**^ **)**	1.12 (0.28)	1.22(0.34)	0.65^a^^`^
**Left atrial diameter (cm)**	5.6 (1.1)	5.91(1.43)	0.22^a^
**Left ventricular Ejection Fraction (%)**	59.4 (7.9)	60.3 (4.8)	0.91^a^
**Echocardiographic Score**			
**Calcification**	1.86 (1.20)	1.84 (1.82)	0.577^a^
**Thickening**	2.00 (1.1)	2.1(1.4)	0.22^a^
**Mobility**	1.68 (0.89)	1.66(0.91)	0.54^a^
**Subvalvular thickening**	1.69 (1.12)	1.71(1.01)	0.59^a^
**Total Echocardiographic score**	13.4 (2.4)	14.3(1.9)	0.30^a^
**Duration of surgery (minutes)**	288 (10.5)	300(5.2)	0.72^a^
**Duration of intubation (minutes)**	840 (12.88)	860(15.65)	0.64^a^
**Cross Clamp Time (minutes)**	63.2(13.7)	64.17(14.09)	0.31^a^
**Bypass time (minutes)**	115(18.5)	111(20.13)	0.12^a^
**Post-operative Medications n (%)**			
**Anticoagulants**	50(100%)	50(100%)	0.24^b^
**Beta-Blockers**	26(52%)	32(64%)	0.82^b^
**Digoxin**	15(30%)	12(24%)	0.99^b^
**Diuretics**	24(48%)	26(52%)	0.23^b^
**Length of stay in ICU(d)**	3.1±0.3	2.9±0.2	0.68^a^
**Total Length of post-operative hospitalization (d)**	10.5±1.4	9.5±1.1	0.12^a^

Continuous data are presented as mean (standard deviation), while categorical data are presented as absolute number (percentage %). BMI: Body mass index; NYHA: New York Heart Association; LVESD: Left ventricular end systolic diameter; LVEDD: Left ventricular end diastolic diameter; PASP: Pulmonary artery systolic pressure; d: Days; P value: Probability value with statistical significance set at the level of P < 0.05.

^a^ P value reported based upon non paired student t-test.

^b^ P value reported based upon Chi-square test.

### Sample size calculation

A Priori sample size calculation was performed using G*Power 3.1.9.4 software (Heinrich-Heine-Universität Düsseldorf, Düsseldorf, Germany; http://www.gpower.hhu.de/). Alpha level was set at α = 0.05, the statistical power was set at 90%; confidence level of 95% and effect size of 0.7 for FVC obtained from our previous quasi-experimental pilot trial with 30 participants (with the outcome measures obtained preoperative and on discharge from hospital), the sample size was calculated as 44 participants per group (total number of 88). Anticipating an attrition rate of 10–15%, so fifty participants were recruited in each group (total sample size n = 100).

### Outcome measures

The primary outcome measures were the pulmonary function measures and the inspiratory muscle strength obtained by measuring maximal inspiratory pressure (MIP). The secondary outcome measure was the functional capacity evaluated by 6-minute walk test (6MWT).

### Procedures

The patients were admitted to the hospital one day before surgery. This is a routine procedure followed for any patient undergoing cardiothoracic surgery for lab investigation, general check-up, pulmonary function testing and pre-operative physiotherapy education. This preoperative education session consisted of instructions about surgery and the importance of physiotherapy program to avoid PPC, training for breathing exercise [Diaphragmatic breathing, segmental breathing], coughing and huffing techniques, ankle pump exercise, lower limb active range of motion (ROM) exercise as well as training for mobility and transfer activities. The purpose of this preoperative education is to help the patients to do these exercise much easier in the postoperative period as they become aware about these exercises in advance, and to improve their awareness about the importance of chest physiotherapy to avoid postoperative complications.

### Evaluation of the studied variables

Assessment of Pulmonary functions and MIP were performed preoperatively (one day before surgery) as a baseline assessment; on the first day in the inpatient ward (POD3) after discharge from the ICU; on the day of discharge from hospital; in the 4^th^ postoperative week; in the 8^th^ post-operative week and after 6 months from surgery (follow-up).

Pulmonary function measurements were performed at rest using a computer-based spirometry system (Eric Jaeger- Germany). Computer-based spirometry system is a valid and reliable method for measurement of pulmonary functions [11]. The patients were asked to be in a relaxed sitting on a height-adjustable chair with arm support (to prevent falling sideways just in case if syncope occurs), with feet supported on the floor, keeping spine erect with shoulders slightly back and using disposable nasal clip and mouth piece for each patient which must be disposed at the end of testing session [12, 13].

The studied spirometry parameters were the forced vital capacity (FVC), forced expiratory volume in one second (FEV_1_), and FEV_1_/FVC ratio. FVC is the amount of air that can be forcibly exhaled from your lungs after taking the deepest breath possible, as measured by spirometry. FEV_1_ is the volume of air that is forcibly exhaled after full inspiration in the first second. FEV_1_/FVC ratio is a calculated ratio used in the diagnosis of obstructive and restrictive lung disease. It represents the proportion of a person’s vital capacity that they are able to expire in the first second of forced expiration to the full, forced vital capacity [13].

The highest value from at least three technically acceptable spirometric maneuvers was recorded. Pulmonary function measurements were performed by an independent examiner who was blinded to the study objectives, group allocations and intervention. This examiner did not participate in the intervention procedures.

Measurement of MIP was performed using a portable electronic respiratory mouth pressure meter device (Micro RPM, Micro Medical Ltd, Kent, UK), which is a reliable and valid tool for measurement of MIP [14]. The measurements were performed while each patient was seated using a nose clip. The patient was instructed to press tightly using his/her lips against the mouthpiece to prevent air leak. The MIP was measured by deep inspiration through the mouthpiece only from the residual volume after maximal expiration. Leaning forward was not allowed during testing as it can overestimate the measurement values [15].

To ensure reproducibility, Testing was repeated with 1-min interval for three technically satisfactory trials with less than 5% difference, and the highest value was used to define MIP expressed in centimeters of water (cmH_2_O). MIP was measured according to the recommendation of American Thoracic Society/European Respiratory Society [16–18].

Functional capacity was examined using six minute walk test (6-MWT). The 6-minute walk test had been clinically validated and it is used to determine the effects of therapeutic interventions and prognosis [19]. The participants were instructed to walk as far as possible without running in six minutes in an enclosed 30-m long hospital corridor. Standardized encouragement was given in every 30 sec, the maximum distance covered at the end of the test was recorded. For safety, Heart rate (HR), respiratory rate, non-invasive arterial blood pressure (ABP), and oxygen saturation were measured before, during and after the test. Heart rate was measured using a continuously monitored electrocardiogram and arterial oxygen saturation was measured by pulse oximetry (Nonin 8500 M; Nonin Medical, Minneapolis, MN, USA). ABP was recorded with the Finometer TM device (FMS, Finapress Measurement Systems, Amsterdam, Netherlands). The test was terminated, if the HR and/or ABP were increases by ≥20% of baseline values.

There is strong evidence of a learning effect for the 6MWT, whereby the second test is usually better than the first. For this reason, having two tests makes intuitive sense to mitigate the influence of this learning bias [20]. Recording the mean value for the two trials is another strategy to avoid the effect of the learning bias, so, the test was performed twice separated by a recovery period of 15 minutes, and then the mean of the two trials was recorded [20–22].

6MWT was performed on the baseline assessment (one day before surgery), on discharge from hospital, 4 weeks from surgery, after 8 weeks from surgery and after 6 months from surgery (Follow up). All assessments (pre-operative & post-intervention) were performed by the same independent examiner.

The patients were admitted to operating theater for conventional mitral valve replacement with sternotomy incision (open-heart surgery), and then referred to the intensive care unit (ICU). After discharge from ICU, the patients were randomly allocated to either experimental (n = 50) group or control group (n = 50). Assessment of pulmonary function was done in the first day of inpatient ward values.

### Postoperative protocol

Both groups received the routine postoperative physiotherapy protocol [Diaphragmatic and segmental breathing exercise, coughing techniques, active cycles of breathing techniques, and active exercise for upper and lower limbs, early ambulation program]. This traditional physiotherapy program was performed twice daily till discharge from the hospital.

In addition to the traditional physiotherapy program, the experimental group received IMT using a pressure threshold loading device (Threshold Inspiratory Muscle Training, Respironics, Pittsburg, PA, USA). This device allows variable loading at a detectable intensity by providing air flow-independent resistance to inspiration using a spring-loaded one- way valve. Changing spring length, results in the same change in the valve opening pressure. This spring-loaded valve opens only when the inspiratory pressure generated by the patients exceeds the spring tension.

At the beginning of the postoperative intervention, the patients started training with 40% MIP recorded pre-operatively. MIP was measured weekly to adjust the training intensity. The intensity was increased incrementally by 5%-10% according to patient tolerance each week targeting 80% of the preoperatively determined MIP by the end of 8^th^ week after surgery. To ensure safety of the training, HR, blood pressure, oxygen saturation and respiratory rate were monitored during training sessions.

Training was performed and supervised by a single physiotherapist whose sole task was only performing the prescribed intervention protocol. This physiotherapist was blinded to the group allocation and the study objectives. The training load was continuously monitored to ensure achievement of the target pressure. The patients received postoperative IMT for 20–30 min/session in the form of six sets, each set consisted of five deep breaths against the IMT device, with a short interval of 1-2minute rest between sets. Training was performed on daily basis, twice per day till discharge from hospital. Training was performed from comfortable sitting position using nose clip in every training session and patients were instructed to inspire through a mouth piece at the desirable training load.

On the day of discharge from hospital, all measurements were repeated for all patients (pulmonary function measurements, maximal inspiratory pressure, and functional capacity). After discharge from the hospital, the patients in the experimental group were instructed to visit the outpatient cardiopulmonary rehabilitation unit for continuing intervention program under supervision and for ensuring that patients were receiving the proper training load. The control group participants were invited to visit the outpatient cardiopulmonary rehabilitation unit to continue the postoperative chest physiotherapy in addition to aerobic exercises as a part of the traditional postoperative rehabilitation protocol which was given for both the experimental and control groups. Training was performed four times per week till the eighth postoperative week. All measurements were repeated in the follow-up after 6 months from date of surgery.

### Statistical analysis

Baseline descriptive statistics were compared using independent t-tests for continuous data and Pearson chi- square analysis for categorical data.

Missing data were remedied using last-observation-carried forward and all analyses were based upon intention-to-treat analysis approach [23]. The Normality of data distribution was analyzed with Shapiro Wilk test, indicating normal distribution of data (P˃0.05). Levin test was used to verify data variance equality. As the data were normally distributed, statistical analysis employed within subjects and between groups (groups X time) repeated measures Analysis of Variance (ANOVA) to investigate the effect of IMT on lung functions and functional capacity. Main effect of intervention; main effect of time as well as the interaction effect between intervention and time were investigated.

The repeated-measures data were checked for sphericity violation using Mauchly’s test, the Greenhouse-Geisser correction was conducted when the sphericity was violated. Pairwise comparison and Post-hoc analyses with Bonferroni correction were conducted when there is significant group-time interaction. Both groups were compared preoperatively (Baseline) with POD3, day of discharge from hospital, after 4 weeks from surgery (4^th^POW) and after 8 weeks post-surgery (8^th^ POW) and after 6 months from surgery.

Effect sizes for significant interaction effects are reported as partial eta squared (η^2^) with the following classification to define small (η2 = 0.01), medium (η2 = 0.06), and, large (η2 = 0.14) effect sizes [24].

## Results

### Baseline characteristics

None significant differences were observed between both groups in terms of their baseline demographic and clinical details as shown in Table 1 (P˃ 0.05).

Descriptive statistics for all the studied variables were presented in Table 2. There were no missing data in the current study.

**Table 2:** Descriptive statistics for pulmonary function measures and functional capacity over time for both study and control groups.

	Baseline	POD3	Discharge	POW4	Pow8	6 months
FEV_1_(L)						
**Control**	3.07±0.39	1.97±0.23	2.26±0.21	2.45±0.20	2.62±0.23	3.19±0.16
**Study**	2.93±0.46	1.75±0.14	2.37±0.32	3.10±0.26	3.71±0.32	4.64±0.20
FVC (L)						
**Control**	2.56±0.14	1.93±0.24	2.49±0.09	2.29±0.06	2.40±0.35	2.57±0.09
**Study**	2.60±0.09	2.32±0.11	2.46±0.11	2.64±0.42	3.64±0.12	3.77±0.11
MIP _(_cmH_2_O)						
**Control**	78.36±6.4	57.96±5.9	62.36±5.8	67.14±5.5	72.16±5.5	75.30±5.6
**Study**	78.78±6.2	58.42±6.1	65.76±6.5	75.88±6.1	89.78±5.1	93.86±2.9
FEV_1_/FVC						
**Control**	73.59±4.1	50.47±3.2	53.9±3.1	59.64±2.8	66.72±3.5	78.8±2.02
**Study**	74.01±3.9	50.79±3.9	58.3±4.6	68.37±4.8	80.94±5.2	91.7±3.33
6MWT(m)						
**Control**	441.4±4.5	-------------	317.5±6.1	354.5±16.2	410.9±10.3	469.9±11.8
**Study**	442.7±5.3	-------------	387.7±14.1	433.1±15.6	509.5±16.6	582.6±19.8

Data are presented as Mean ±standard deviation. FVC: Forced vital capacity; FEV_1:_ forced expiratory volume at first second; FEV_1_/FVC: Ratio of forced expiratory volume in the first second to the forced vital capacity; MIP: Maximal Inspiratory Pressure; 6MWT: Six Minute Walk test

### Pulmonary function & MIP

Repeated measure ANOVA revealed significant group-time interaction effect on FVC [F (5,94) = 18.63; P <0.001], FEV_1_ [F(5,94) = 228.15; P <0.001], FVC/FEV_1_ [F(5,94) = 255.06; P <0.001] and MIP [F(5,94) = 183.66; P <0.001] with large effect sizes (η^2^˃ 0.14) as shown in Table 3. These results indicate significant effect of intervention (IMT) on the studied spirometric parameters.

**Table 3:** Repeated measure ANOVA within subjects and between groups comparison.

	Between Groups			Within groups (time main effect)			Group-time interaction		
	F	P value	Effect size	F	P value	Effect size	F	P value	Effect size
**FVC**	15.47	.000*	0.15	752.19	.000*	0.88	18.63	.000*	0.15
**FEV**_**1**_	41.84	.000*	0.29	715.49	.000*	0.88	228.15	.000*	0.71
**FVC/FEV**_**1**_	59.66	.000*	0.38	776.32	.000*	0.97	255.06	.000*	0.69
**MIP**	36.68	.000*	0.27	594.71	.000*	0.85	183.66	.000*	0.65
**6MWT**	125.40	.000*	0.69	1260.91	.000*	0.82	336.12	.000*	0.83

FVC: Forced vital capacity; FEV_1:_ Forced expiratory volume at first second; FEV_1_/FVC: Ratio of forced expiratory volume in the first second to the forced vital capacity; MIP: Maximal Inspiratory Pressure; 6MWT: Six Minute Walk test; P value: Probability value with statistically significant level set at P<0.05

*: Statistically significant.

No significant differences were detected between groups before intervention (at baseline and POD3) (P ˃0.05) in all the studied spirometric parameters. Statistically Significant differences were found between groups in the studied spirometric parameters FVC [F(1,98) = 15.47; P <0.001], FEV_1_ [F(1,98) = 41.84; P <0.001], FVC/FEV_1_[F(1,98) = 59.66; P <0.001] with large effect size (η^2^ ˃ 0.14). Furthermore, statistically significant differences were observed in all the studied spirometric parameters within groups over time (P <0.001) as displayed in Table 3.

Post hoc analysis using Bonferroni adjustment for pairwise comparisons was used to detect at which time point the significant differences were found. Post-hoc analysis revealed statistically significant differences between groups (P <0.05) in the studied spirometric parameters (FVC, FEV_1_, FEV_1_/FVC) and MIP at the following time points [discharge from hospital, after 4 weeks from date of surgery (POW4), and after 8 weeks from date of surgery (POW8)]. There were no statistically significant differences between groups (P˃0.05) in the studied spirometric parameters as well as MIP at baseline assessment and in the third postoperative day (POD3) after discharge from ICU.

Pairwise comparison within groups indicates significant differences over time compared to the baseline measurements in both groups (P<0.05). Compared to the baseline assessment, all values of the studied spirometric parameters and MIP showed sharp reduction after surgery (POD3) before starting the protocol of intervention.

**Fig 2:**
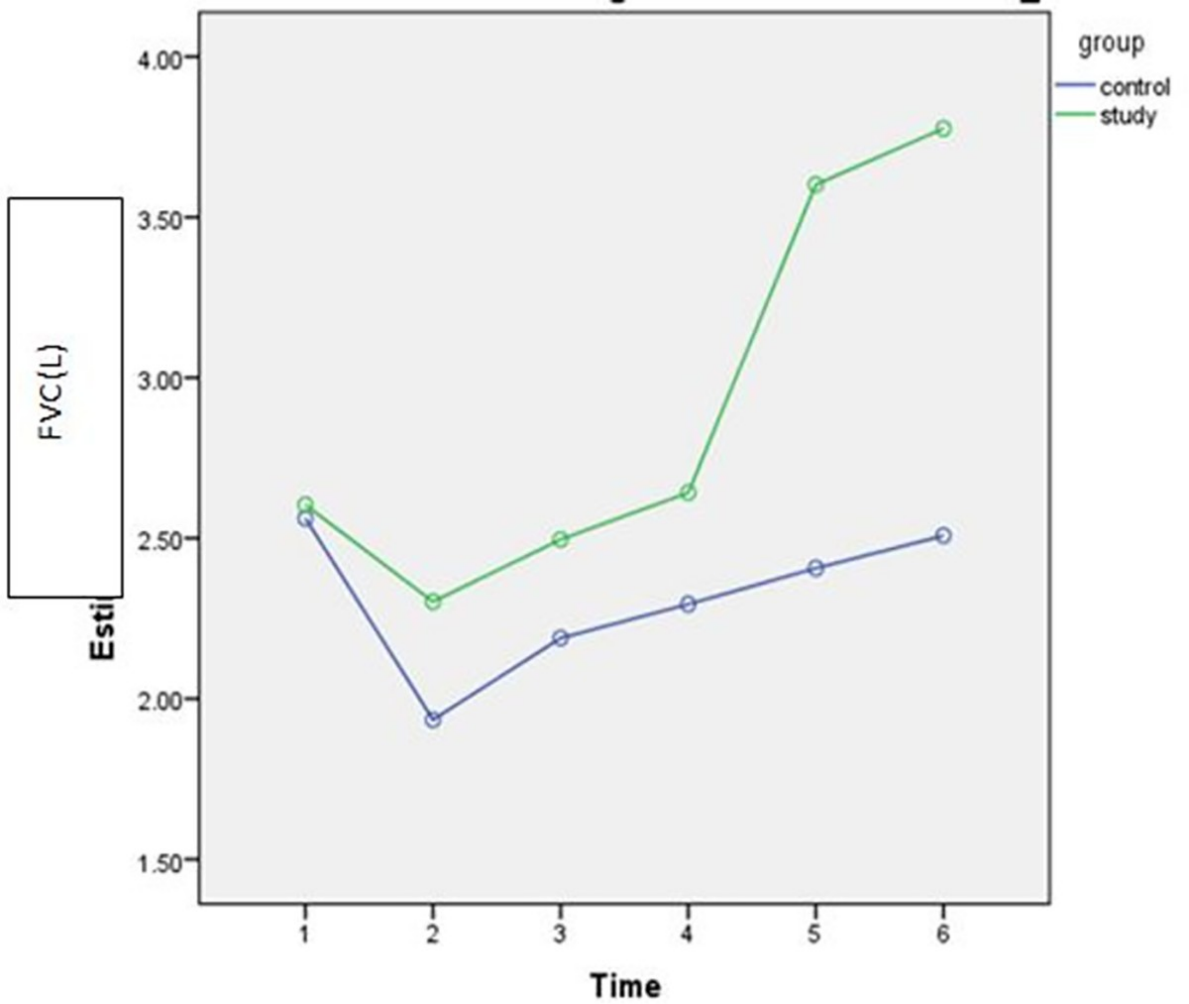
Estimated marginal means of FVC [1: Pre-operative; 2: POD3; 3: Discharge; 4: 4^th^ POW; 5: 8^th^POW; 6: Follow-up].

**Fig 3:**
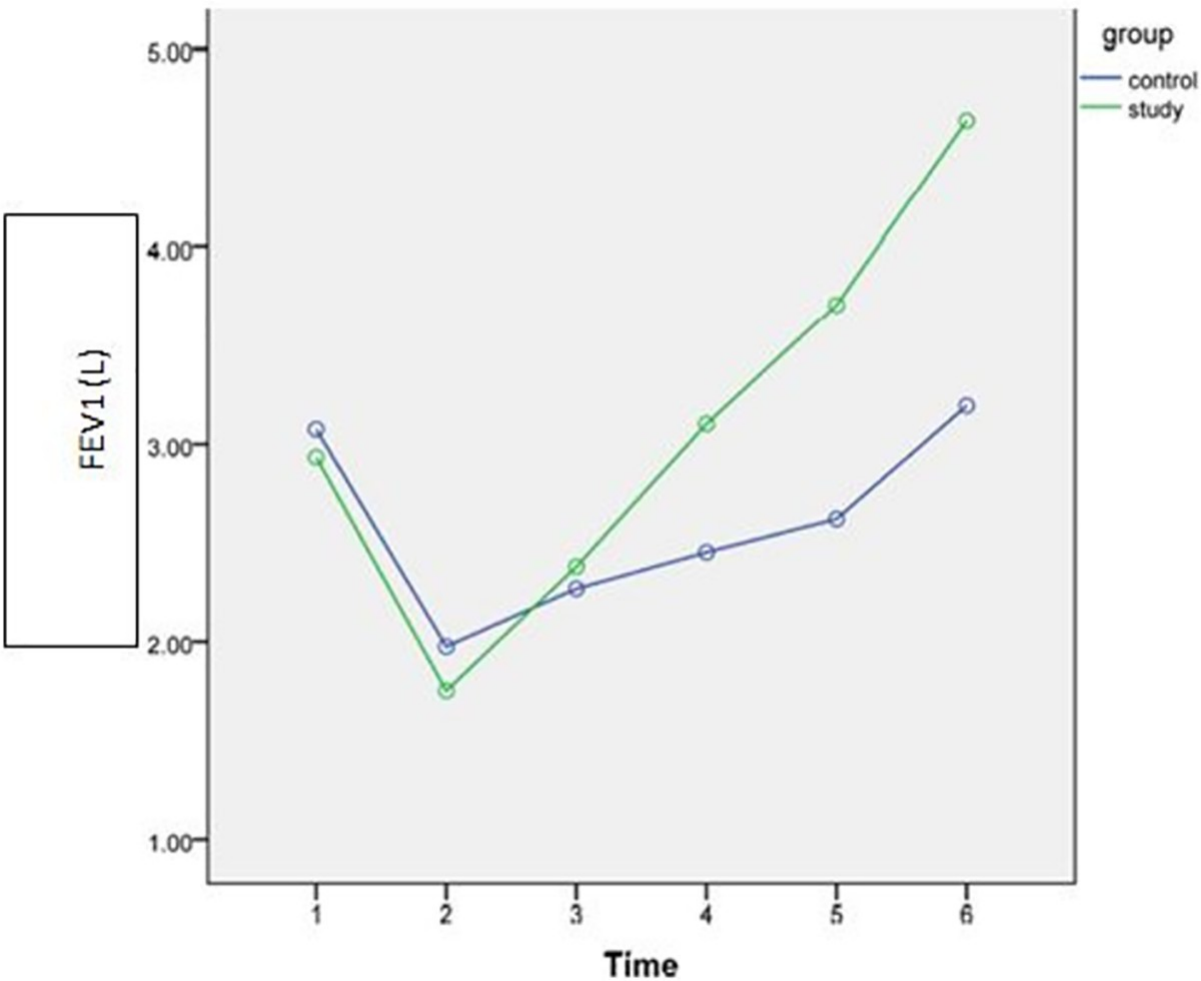
Estimated marginal means of FEV_1_ [1: Pre-operative; 2: POD3; 3: Discharge; 4: 4^th^ POW; 5: 8^th^POW; 6: Follow-up].

**Fig 4:**
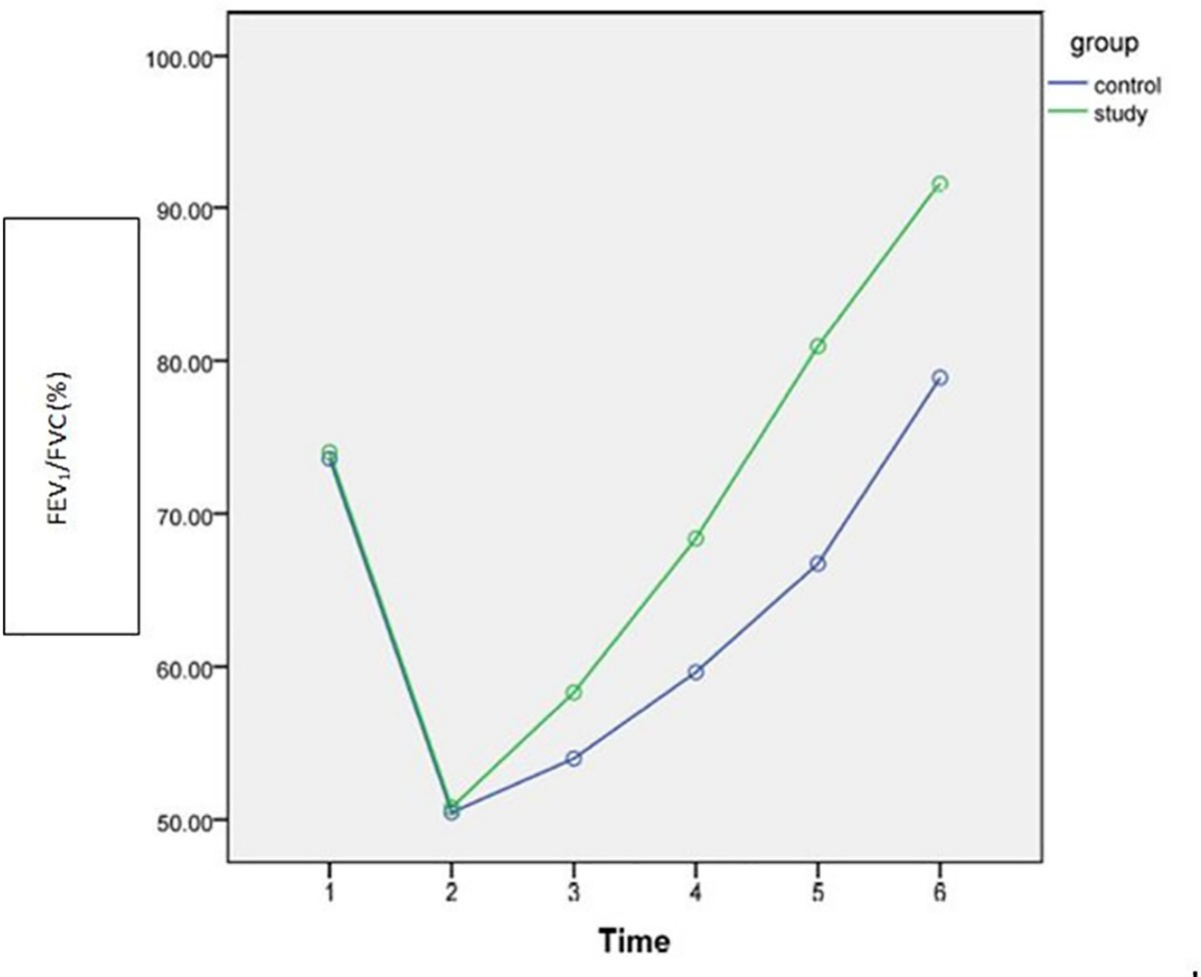
Estimated marginal means of FEV_1_ /FVC [1: Pre-operative; 2: POD3; 3: Discharge; 4: 4^th^ POW; 5: 8^th^POW; 6: Follow-up].

**Fig 5:**
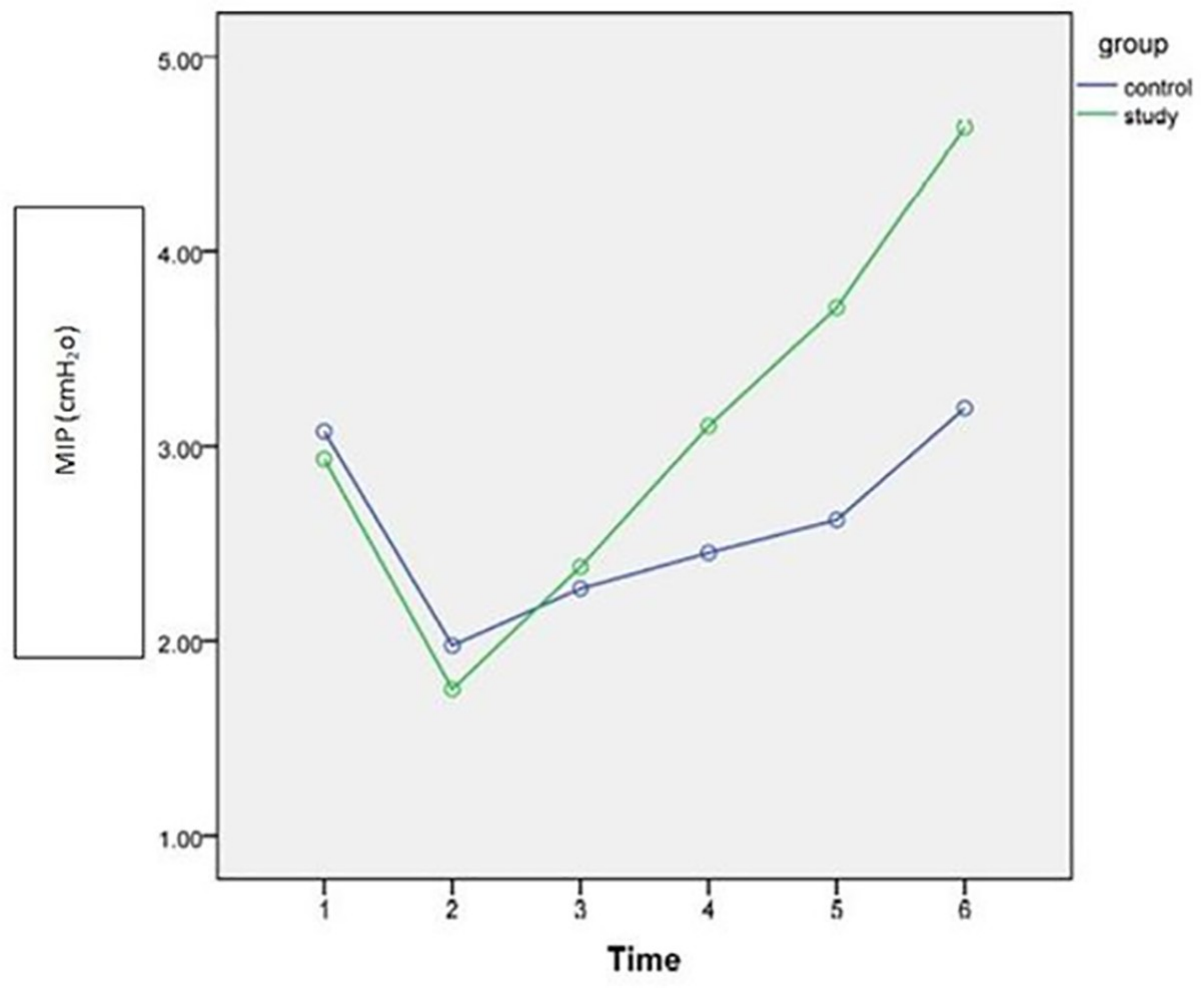
Estimated marginal means of MIP [1: Pre-operative; 2: POD3; 3: Discharge; 4: 4^th^ POW; 5: 8^th^POW; 6: Follow-up].

Gradual improvements were observed after intervention over time (discharge, POW4, POW8) in both study and control groups as shown in Figs 2–5. However, the rate of improvement over time in the control group was lower than in the study group. In the eighth post-operative week, the values of all spirometric parameters in the study group were significantly higher than baseline values (Table 2) and significantly different than the values in the control group (P<0.001) as presented in Table 4. This significant difference also was apparent at the follow up assessment (6 months after surgery) after stopping the intervention.

**Table 4:** Pairwise comparison between study (n = 50) and control groups (n = 50) over time.

	Base line					POD3					Discharge					POW4					POW8					6 Months after surgery				
	MD	SE	Sig^b^	95% CI		MD	SE	Sig^b^	95%CI		MD	SE	Sig^b^	95%CI		MD	SE	Sig^b^	95%CI		MD	SE	Sig^b^	95%CI		MD	SE	Sig^b^	95%CI	
				LB	UB				LB	UB				LB	UB				LB	UB				LB	UB				LB	UB
FVC (L)	.042	.024	.08	.006	.090	.37	.03	.02	0.29	0.44	.307	.02	.00*	0.272	0.341	.347	.06	.00*	0.23	0.46	1.20	.02	.00*	1.15	1.23	1.27	.036	.00*	1.14	1.393
FEV_1_ (L)	.142	.086	.10	.029	.314	.22	.04	.10	0.14	0.30	.111	.06	.04*	0.003	0.220	.650	.05	.00*	0.55	0.74	1.28	.08	.00*	0.97	1.20	1.45	.037	.00*	1.37	1.522
FEV_1_ /FVC _(%)_	.420	.812	.60	-1.1	2.03	.33	.72	.65	-1.11	1.76	4.31	.80	.00*	2.74	5.89	8.74	0.8	.00*	7.15	10.3	14.3	.90	.00*	12.43	15.99	12.8	.775	.00*	11.6	13.86
MIP (cmH_2_O)	.420	1.26	0.74	.022	1.54	.46	1.2	.72	0.15	1.93	3.48	1.25	.00*	0.924	5.87	8.74	1.18	.00*	6.40	11.1	17.6	1.1	.00*	15.50	19.73	18.6	.898	.00*	16.7	20.34
6MWT (m)	1.300	.977	.186	-.63	3.24	----	----	---	-----	-----	70.3	2.17	.00*	65.93	74.55	78.6	3.18	.00*	72.2	84.8	98.6	2.8	.00*	93.08	104.03	112.8	3.25	.00*	106.3	119.2

FVC: Forced vital capacity; FEV_1:_ forced expiratory volume at first second; FEV_1_/FVC: Ratio of forced expiratory volume in the first second to the forced vital capacity; MIP: Maximal Inspiratory Pressure; 6MWT: Six Minute Walk test; POD3: First day in the inpatient ward after discharge from ICU; POW4: Postoperative 4^th^ week; POW8: Postoperative 8^th^ week; MD: Mean difference; SE: Standard error. 95%CI: 95% confidence interval; LB: Lower border; UB: Upper border.

*. The mean difference is significant at the level of P <0.05

b. Adjustment for multiple comparisons using Bonferroni test in post-hoc analysis

Sig: Significance level.

### Functional capacity

The current study revealed a significant group-time interaction effect on functional capacity reflected by 6MWT [F(4,95) = 336.12; P <0.001], that was justified by large effect sizes (η^2^ = 0.83) as shown in Table 3. Statistically Significant difference was found between groups in 6MWT [F (1,98) = 125.40; P <0.001] with large effect size (η^2^ = 0.69). Similarly, statistically significant difference was observed within groups over time [F(1.98) = 1260.91; P<0.001] with large effect size (η^2^ = 0.83). These results indicate effectiveness of IMT on functional capacity (Table 3).

**Fig 6:**
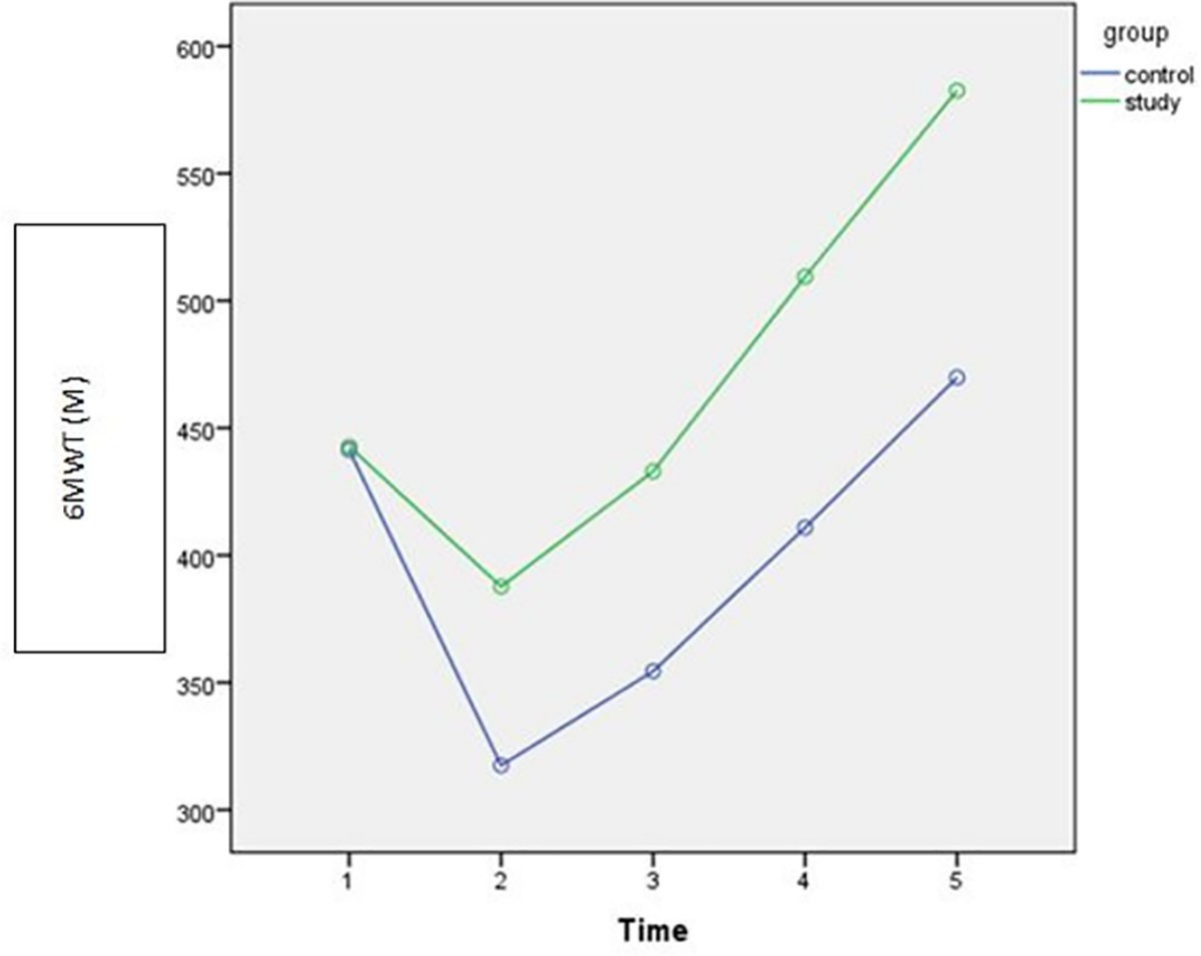
Estimated marginal means of functional capacity measured by (6MWT) [1: Pre-operative; 2: Discharge; 3: 4^th^ POW; 4: 8^th^POW; 5: Follow-up].

Following the successful repeated measures ANOVA, the Post-hoc analysis revealed a non-statistically significant difference in functional capacity quantified by 6MWT between groups before intervention at the baseline (pre-operative) assessment (P˃0.05). However, statistically significant differences were found between groups after intervention (P <0.05). The experimental group showed significant improvement in the distance measured by 6MWT over time (P <0.001). By the end of intervention protocol (8 months), the functional capacity in the experimental group was significantly higher than the baseline assessment which was not seen in the control group. This improvement lasted after stopping the intervention at follow up (6months) as shown in Fig 6 and presented in Table 4.

## Discussion

The current study was designed to investigate the effect of postoperative high intensity IMT for eight weeks on the pulmonary function, inspiratory muscle strength and functional capacity after MVRS. The results of the current study revealed that postoperative high load IMT improves pulmonary functions, inspiratory muscle power and functional capacity significantly higher than the traditional postoperative cardiopulmonary rehabilitation protocol. That does not mean ineffectiveness of the traditional postoperative rehabilitation protocol; however, the rate of improvement in the control group was obviously much lower than that in the experimental (IMT) group as discussed in the following section.

### Effect of IMT on pulmonary functions

The results of the current study revealed that IMT improved lung function, returning almost to the pre-operative level after four weeks from date of surgery, and improved to above the pre-operative level in the eighth postoperative week in the experimental group. The control group did not show the same progress as in experimental group, as the lung function were still below preoperative level by the end of eighth postoperative week. This significant differences between experimental and control group were justified by large effect size (η^2^ ˃ 0.14).

These observations in the control group are consistent with the previous studies which reported that the lung function values returned to the preoperative level after four months from surgery [25, 26]. Patients undergoing cardiac surgery develop restrictive lung volumes, impaired ventilatory mechanics, decreased lung compliance and increased breathing effort postoperatively. These impairments in lung functions are caused by many factors affecting the chest wall mechanics such as sternotomy incision, diaphragmatic paresis due to cooling of pericardium, rib injury, trauma to costochondral junction, sternal instability and violation of the pleural space [7].

On the other hand, experimental group showed significant improvement in pulmonary function after IMT program reflected by significant increase in the spirometric parameters (FVC, FEV_1_ and FEV1/FVC). These results were consistent with the previous study by Cargnin et al. [9], who investigated the effect of post-operative IMT after heart valve replacement for 4 weeks. They reported that spirometry parameters at the end of training were almost the same as the pre-operative level.

The observed improvement in pulmonary function after IMT could be explained by restoring the respiratory muscle strength through improving maximal inspiratory pressure (MIP) and endurance and improving the tidal volume and peak expiratory flow which improves the ability to cough as well as maintaining airway patency leading to better prognosis and reducing PPC [3, 5, 27]. Moreover, IMT induces an improvement in the size of type II muscle fibers of the inspiratory muscles leading to increase in the shortening velocity of these muscles, allowing more time for expiration and reducing lung hyperinflation [15, 28].

The results of the current study indicated that 4 weeks of post-operative IMT were enough to restore the lung function to almost the pre-operative level, while, 8 weeks of IMT post-operatively yields a significant improvement in the lung function much higher than the pre-operative level. This improvement in the experimental group was justified by remaining higher than the control group for 6 months after surgery in the follow up measurement.

### Effect of IMT on inspiratory muscle strength

The inspiratory muscles strength is quantified by (MIP) [7]. The current study results revealed that MIP demonstrated sharp drop of up to 62% in both control and experimental groups during the first post-operative week after MVRS. These findings come online with the previous studies that reported reduction of MIP by 60% in the first week after cardiac surgery [9, 29, 30], and may take up to 6–8 weeks to reverse [31, 32]. This reduction in MIP is explained by respiratory suppression due to anesthesia, postoperative pain, diaphragmatic dysfunction and sternotomy incision which reduces thoracic mobility.

The experimental group showed gradual recovery of MIP after the first postoperative week till reaches closer to the pre-operative level by the end of the first post-operative month. By the end of 8^th^ postoperative week, the experimental group presented a significant increase in MIP, which was much higher than the pre-operative level and higher than the control group as well. On the other hand, MIP in the control group recovered to a level closer to the pre-operative level by the end of IMT (8 weeks post-surgery).

The improvement of MIP in the experimental group can be explained by improved inspiratory muscle strength and endurance induced by IMT due to improving the neuromuscular recruitment pattern resulting from repeated exposure to the same task (additional resistance to the inspiration process) [33]. Thus, IMT improves the inspiratory muscle strength leading to improving oxygenation and reduce the risk of developing PPC, the length of hospital stay and the cost of hospitalization consequently.

The current study results were supported by the previous systematic review and meta-analysis [3, 7] and were consistent with the previous study by Cargnin et al. [9], which reported improvement of MIP to almost the pre-operative level in IMT group only. However, in their study [9] the training protocol was only 4 weeks and the participants’ age rang was wider (20–80 years old) than the current study. Moreover, they did not investigate the long term effect of IMT through follow up as the current study did.

Furthermore, the current study results contradict with the previous study by Cordeiro et al. [5], who reported that IMT post-operatively till discharge from hospital was enough to restore the MIP to the pre-operative level after cardiac surgeries. The differences between their study and the current study in the sample characteristics, sample size (n = 50), and the methodology followed (unclear prescription of IMT dose), make the comparison difficult.

The improvement in MIP in the experimental group lasted for 6 months after surgery as presented in the follow up which provide evidence about the effectiveness of IMT on improving inspiratory muscle strength. This study come online with the previous study by Martin-Sanchez et al. [33], who reported that high load of IMT (40%MIP) achieved higher improvement in the MIP than lower load IMT (20%) in institutionalized elderly women after 8 weeks of training.

### Effect of IMT on functional capacity

In the current study, the functional capacity assessed by the 6MWT, returned almost to the pre-operative level after 4 weeks of the post-operative IMT in the experimental group. By the end of intervention protocol of IMT, the experimental group demonstrated significant improvement in functional capacity much higher than the pre-operative level. This progress was not seen in the control group, which hardly recover the functional capacity to the pre-operative level by the end of the 8^th^ weeks after surgery.

These results could be explained by improving the inspiratory muscle strength and endurance after IMT, which improves oxygenation and increase oxygen delivery to the limb muscles consequently, leading to less fatigue and improve functional capacity [9, 34, 35]. These results agree with the previous studies [9, 34], which reported improvement in functional capacity with post-operative IMT after pulmonary resection and after cardiac surgeries.

The improvement in functional capacity in the experimental group lasted for 6 months after surgery as presented in the follow up after 6 months from surgery, which justify the effectiveness of IMT on improving inspiratory muscle strength. This improvement in functional capacity was not observed in the control group.

### Clinical significance

This study has valuable clinical implications as it presents promising clinical findings regarding the effect of using high intensity IMT for long duration (8 weeks). Using these parameters of IMT, resulted in faster recovery of the pulmonary functions, inspiratory muscle strength and functional capacity after mitral valve replacement compared to the traditional protocol. Moreover, the results revealed that using high intensity IMT (more than 40% MIP) is a safe intervention in the early post-operative period after MVRS. Our findings highlighted and recommended that using IMT with staring load of 40% of the pre-operative IMT targeting 80% MIP for long duration (8 weeks) are effective parameters of the post-operative IMT to be used clinically as there were a paucity of literature regarding this point [3, 7]. This will have positive impact on improving and upgrading the evidence based clinical practice.

### Strengths of the study

The majority of the previously published studies investigated the effect of pre-operative IMT on pulmonary function and functional capacity. However, there is a shortage of the available literature regarding its effectiveness when carried out post-operatively [3, 7]. The strength points of the current study are as following:

- This is the first study to investigate and provide an evidence about the effectiveness of post-operative high load long duration IMT on pulmonary function and functional capacity after mitral valve replacement surgeries
- The study design (prospective randomized controlled trial with follow up) helps clinicians and researchers to track the progress and changes in pulmonary functions, inspiratory muscle strength and functional capacity over a long period of time. Furthermore, The follow up assessment provide an evidence about the long term effect of high load, long duration IMT
- Being randomized trial with monitoring the compliance to the intervention, reduces the risk of bias and improves the generalizability of the obtained results

### Limitation

The current study didn’t investigate the effect of different doses of IMT (ie, intensity, frequency, duration of the training protocol) and timing of the intervention (pre-operative, post-operative or combination) on the pulmonary function, inspiratory muscle strength and the functional capacity. This should be addressed in the future studies, because of paucity of the available literature regarding the most effective IMT protocols [7]. Furthermore, investigating the preventive effect of IMT on developing the PPC was beyond the scope of the current study and should be addressed in the near future studies. Finally, the current study did not investigate the progress of post-operative cardiac function over time based upon echocardiography findings, because it was not one of the current study objectives.

## Conclusion

High load IMT (more than 40% MIP) for 8 weeks should be included as a basic part in the post-operative cardiopulmonary rehabilitation as it is safe and it seems promising to improve lung function, inspiratory muscle strength and functional capacity. So, it is recommended to be used as a basic part in the postoperative cardiopulmonary rehabilitation after MVRS.

## Supporting information

S1 Checklist CONSORT checklist. (DOC)

S1 File Study protocol. (DOCX)
