# Supplementary material for: Effect of postoperative high load long duration inspiratory muscle training on pulmonary function and functional capacity after mitral valve replacement surgery: A randomized controlled trial with follow-up
Source: PLoS One. 2021 Aug 27;16(8):e0256609. doi: 10.1371/journal.pone.0256609 (PMC8396720; doi:10.1371/journal.pone.0256609)
Supplement: S1 File — (DOCX) [file pone.0256609.s002.docx]

**Research Proposal**

**Effect of high load long duration postoperative inspiratory muscle training on pulmonary function and functional capacity after mitral valve replacement surgery: A randomized controlled trial**

**Submitted to**

**Human Research Ethics Committee of South Valley University**

April 2019

**Introduction:**

Mitral valve replacement is a cardiac surgical procedure in which the diseased mitral valve is replaced by a mechanical or biological tissue valve.

Pulmonary physiology and mechanics are further disturbed after cardiothoracic surgery. Recovery from major surgery especially from open heart surgery is mainly affected by postoperative pulmonary complications (PPCs). The incidence of postoperative pulmonary complications is more frequent in upper abdominal and thoracic surgeries[1].

Cardiac Surgery may affect respiratory muscle function by a number of different mechanisms. For example, surgical incision of the chest wall affects the integrity of the respiratory muscles and provoke both alteration in demand and availability of energy for contraction of these muscles and thus directly affects their function leading to a reduction in the vital capacity (VC), tidal volume (VT), total lung capacity (TLC) and, thus, insufficient cough. Which in turn cause atelectasis in the basal lung segments and a decrease in functional capacity (FRC)[2]**.**

In any major surgery, like, thoracic, or abdominal surgery, weighing benefits and risks is crucial for decision. With regard to risks arising from surgery, the prevention of postoperative pulmonary complications (PPC) is relevant, as they are strongly associated with increased morbidity and mortality, and decreased quality of life. The most common PPC are atelectasis, pneumonia, bronchospasm, embolus, acute respiratory distress syndrome, respiratory failure with prolonged mechanical ventilation, pleural disorders and diaphragmatic and/or chest wall dysfunction [1,3–5]. PPC seem to play an important role in length of hospital stay (LOS) and treatment costs [1,2,6].

Inspiratory muscle training (IMT) might be an important strategy to reduce PPC and LOS after major surgery, especially those involving respiratory muscle and chest wall damage. Aiming at elucidating the potential benefits of IMT on PPC and LOS, several meta-analyses have been conducted, but all analysing only preoperative IMT[3–6].

In their systematic review, Kendal et al.,[1] reported that there is paucity of the available literature investigating the effectiveness of postoperative IMT and its proper dose prescription [ie, starting load, maximal achieved load, Load increment, intervention duration, frequency, duration of sessions and the degree of supervision], which makes it difficult to ascertain the effectiveness and the preventive ability of IMT in reducing the risk of developing PPC.

Few studies had investigated the effectiveness of postoperative IMT with different methodologies, small sample size, different protocols, different durations of intervention with some supporting one week of intervention[7], while others investigated the effect of two weeks of interventions[8]. Different methodologies and unclear IMT parameters makes it difficult to compare between previous studies and consequently difficult to make clinical recommendation for using the postoperative IMT.

Pulmonary function values measured by computerized spirometry is simple and safe investigation routinely used in clinical practice for the diagnosis and evaluation of a variety of pulmonary diseases. The most important of these tests include spirometry. Pulmonary function values are based on sex, heights, weight and age. When the patient performs the test, actual results (observed) will be compared with the predicted values expected of a person of gender, height, and age to see if he falls within the "normal" range, or has a restrictive or obstructive component based on the results of the tests [9].

The Six minute walk test (6MWT) is used to measure cardiovascular endurance and functional capacity following abdominal and cardiothoracic surgery. Spirometry is a gold standard outcome measure, used to assess pulmonary function[10]. Spirometry and 6MWT are excellent tests for assessing pulmonary function and functional capacity before and after open abdominal and cardiothoracic surgery[11]

**Statement of the problem:**

Does postoperative high load long duraion inspiratory muscle training (IMT) affect pulmonary function and functional capacity after Mitral valve replacement surgery?

**Purpose of the study:**

The purpose of the study was to investigate the effect of the postoperative high load long duration inspiratory muscle training on pulmonary function and functional capacity after mitral valve replacement.

**Significance of the study:**

Reductions in oxygenation, pulmonary function, and respiratory muscle strength, as well as radiological changes such as atelectasis have been cited as common alterations in postoperative cardiac surgery. The reduction of Respiratory Muscle Strength (RMS), resulting from direct or indirect lesion of respiratory muscles during surgery and the secondary diaphragmatic dysfunction due to phrenic nerve lesion, has also been related to reduced pulmonary function tests, worsened gas exchange, and increase in the rate of pulmonary complications [12]**.**

It was reported that high load pre-operative IMT was related to reduction in PPC[1,13] however, to the best of our knowledge, there is no study had investigated the effectiveness of using different doses of IMT postoperatively. Since, the benefits of IMT was related to the prescribed dose[1], this study was designed to investigate the effect of high load long duration (8 weeks) supervised IMT on pulmonary function, and functional capacity after mitral valve replacement. That will investigate the effectiveness of IMT dose (High load , long duration, supervised training) on pulmonary function and functional capacity. Furthermore, the study design (prospective randomized controlled trial with follow up) will help clinicians and researchers to track the progress and changes in pulmonary functions, inspiratory muscle strength and functional capacity over a long period of time after Mitral valve surgery. Furthermore, the follow up assessment will provide an evidence about the long term effect of high load IMT

**Hypotheses:**

It was hypothesized that there will be an effect of postoperative IMT on pulmonary function after mitral valve replacement

**REVIEW OF THE RELATED LITERATURE**

Mitral valve disease patients may exhibit reductions in lung volumes due to inspiratory muscle weakness in cases of pulmonary vascular congestion. The inspiratory muscles are compromised due to poor nutritional status that lead to decreased muscle mass and strength, increased work of breathing, in cases of long-standing mitral valve disease [14,15].

Pulmonary hypertension develops in these patients also, as a result of combination of transmission of raised left atrial pressure, pulmonary arteriolar vasoconstriction and obliterative changes in the pulmonary circulation [16].

The incidence of postoperative pulmonary complications is more common in upper abdominal and cardiothoracic surgeries and may include pleural effusion, atelectasis, pneumothorax, bronchospasm, respiratory infection, respiratory failure, pneumonia, acute respiratory distress syndrome (ARDS), and pulmonary embolism [17].

The cardiac surgeries predispose to abnormal changes in respiratory mechanics, lung volumes and gas exchange, leading to development of postoperative respiratory changes. These changes are due to various causes [such as the condition of the heart and lung preoperatively, the degree of sedation, intensity of surgical manipulation and the number of pleural drains] which leads to development of abnormal respiratory mechanics in the immediate postoperative period [18,19].

Mitral valve surgery causes insufficient diaphragmatic breathing post operatively which contributes significantly to development of postoperative pulmonary complications. Consequently, there are consistent reductions in pulmonary volumes and flows, alveolar collapse, reduction of sputum clearance ability, increase of respiratory work and diminution of mechanical efficiency of the respiratory muscles [3].

Inspiratory muscle training is one of the procedures followed to promote better efficacy in airway clearance, improving inspiratory pressure and prevent fatigue of the respiratory muscles [20], which aims to improve functional capacity, respiratory muscle strength and reduce imminent risks of developing post-operative complications in patients undergoing cardiovascular surgery [21].

In phase I of Cardiac Rehabilitation various physiotherapy techniques are routinely used such as diaphragmatic breathing exercises, effective cough, chest wall percussion and vibration, continuous Positive Airway Pressure (CPAP) may prevent further deterioration in pulmonary function and reduce the incidence of Post-operative pulmonary complications[17,22,23]. Unfortunately, There is a controversy about the efficacy of these postoperative procedures in decreasing the incidence of Post-operative pulmonary complications and improving pulmonary flows and volumes [4].

Inspiratory muscle training (IMT) is a therapeutic tool and one component of pulmonary rehabilitation which improves respiratory muscle strength, reduce the severity of dyspnea, improve the exercise tolerance and improve pulmonary flows and volumes after cardiothoracic surgery [4,24].

**MATERIALS AND METHODS**

**Design of the study:**

Randomized controlled trial

**Sample size calculation:**

A Priori sample size calculation was performed using G*Power 3.1.9.4 software (Heinrich-Heine-Universität Düsseldorf, Düsseldorf, Germany; <http://www.gpower.hhu.de/>). Alpha level will be set at α = 0.05, the statistical power was set at 90%; confidence level of 95% and effect size of 0.7 for FVC obtained from our previous pilot study, the sample size was calculated as 44 participants per group (total number of 88). Anticipating an attrition rate of 10-15%, so fifty participants will be recruited in each group (total sample size n=100).

**Outcome measures:**

The primary outcome measures will be the pulmonary function measures and the inspiratory muscle strength obtained by measuring maximal inspiratory pressure (MIP).The secondary outcome measure will be the functional capacity evaluated by 6-minute walk test (6MWT).

**Participants:**

All participants will be patients aged 25-50 years recruited from the waiting list for elective Mitral valve replacement (MVRS) at South Valley university hospital.

The inclusion criteria are:

1. patients undergoing elective MVRS
2. participants aged 25-50 years
3. normal body mass index (BMI)
4. post-operative medically and clinically stable
5. Able to walk independently without any assistive devices.

The exclusion criteria are:

1) Obesity (BMI ˃ 30kg/m^2^)

2) Postoperative hemodynamic complications such as lung congestion or myocardial infarction

3) History of pulmonary diseases or infection such as chronic obstructive pulmonary diseases (COPD) or tuberculosis

4) Smokers

5) Post-operative renal failure

6) Cardiac arrhythmia or unstable angina

7) Uncontrolled hypertension

8) Post-operative hemodynamic instability

9) Post-operative prolonged ventilation [more than 24 hours]

10) Neurological/ musculoskeletal disorders that could affect functional capacity or pulmonary function such as(parkinsonism, ankylosing spondylitis, scoliosis, ….etc)

11) History of previous cardiothoracic surgery.

**Study Setting:**

South Valley University Hospital; Qena; Egypt

Anticipated trial start date: Jun 2019

Anticipated last follow up date: January 2020

**Randomization, allocation concealment:**

The patients will be randomly selected using simple randomization method. After surgery, random allocation of patients will be performed through sequentially numbered sealed opaque envelops. The sequentially numbered envelops will be arranged and sealed by an independent statistician who will not be involved in the study. These envelops will be given to the allocator. The allocator will be blinded to the study objectives and will not participate either in the intervention or the assessment procedures. The allocation ratio will be 1:1. Each envelop will be opened only in front of the patient to be assigned. Fifty participants were randomly allocated to each of the experimental group (n=50) and control group (n=50).

**Procedures:**

The patients will be admitted to the hospital one day before surgery. This is a routine procedure followed for any patient undergoing cardiothoracic surgery for laboratory investigation, general check-up, pulmonary function testing and pre-operative physiotherapy education. This preoperative education session consisted of instructions about surgery and the importance of physiotherapy program to avoid PPC, training for breathing exercise [ Diaphragmatic breathing, segmental breathing], coughing and huffing techniques, ankle pump exercise, lower limb active range of motion (ROM) exercise as well as training for mobility and transfer activities. The purpose of this preoperative education is to help the patients to do these exercises much easier in the postoperative period as they become aware about these exercises in advance.

Assessment of Pulmonary function, MIP will be performed preoperatively (one day before surgery) as a baseline assessment, on the first day in the inpatient ward, on the day of discharge from hospital, by the end of the first postoperative month, by the end of 8^th^ postoperative week and after 6 months from date of surgery (Follow up). Pulmonary function measurements will be performed at rest using a computer-based spirometry system (Eric Jaeger- Germany). The measurement will be done from a relaxed sitting on a height adjustable chair with arm support (to prevent falling sideways just in case if syncope occur), with feet supported on the floor, keeping spine erect with shoulders slightly back and using disposable nasal clip and mouth piece for each patient which must be disposed at the end of testing session.

The studied spirometry parameters in this study are the forced vital capacity (FVC), forced expiratory volume in one second (FEV_1_), and FEV_1_/FVC ratio. The highest value from at least three technically acceptable spirometric maneuvers will be recorded. Pulmonary function measurements will be performed by an independent examiner who will be blinded to the study objectives, group allocations and intervention. This examiner will not participate in the intervention procedures.

Measurement of MIP will be performed using a portable electronic respiratory mouth pressure meter device (Micro RPM, Micro Medical Ltd, Kent, UK). The measurements will be performed from comfortable sitting position with a nose clip. The MIP is measured by deep inspiration through the mouthpiece only from the residual volume after maximal expiration. Leaning forward will not be allowed during testing as it can overestimate the measurement value.

To ensure reproducibility, Testing will be repeated with 1-min interval for three technically satisfactory trials with less than 5% difference, and the highest value will be used to define MIP expressed in centimeters of water (cmH_2_O).

Functional capacity will be examined using a six minute walk test (6-MWT). The participants will be instructed to walk as far as possible without running in six minutes in an enclosed 30-m long hospital corridor. Standardized encouragement will be given every 30 sec, the maximum distance covered at the end of the test will be recorded. For safety, Heart rate (HR), respiratory rate, blood pressure (ABP), and oxygen saturation will be measured before, during and after the test. The test will terminated, if the HR and/or ABP increases by ≥20% of baseline values. The test will be performed twice separated by a recovery period of 15 minutes, and then the mean of the two trials will be recorded.

The test will be performed on the baseline assessment (one day before surgery), on discharge from hospital, 4 weeks from surgery, after 8 weeks from surgery and after 6 months from surgery (Follow up).

Both groups will receive the routine postoperative physiotherapy protocol [Diaphragmatic and segmental breathing exercise, coughing techniques, active cycles of breathing techniques, and active exercise for upper and lower limbs, early ambulation program]. This traditional physiotherapy program will be performed daily till discharge from the hospital.

In addition to the traditional physiotherapy program, the experimental group will receive IMT using a pressure threshold loading device (Threshold Inspiratory Muscle Training, Respironics, Pittsburg, PA, USA). This device allows variable loading at a detectable intensity by providing air flow-independent resistance to inspiration using a spring-loaded one way valve. Changing spring length results in the same change in the valve opening pressure. This spring-loaded valve opens only when the inspiratory pressure generated by the patients exceeds the spring tension.

At the beginning of the postoperative intervention, the patients started training with 40% MIP recorded pre-operatively. MIP will be measured weekly to adjust the training intensity. The intensity will be increased incrementally by 5%-10% according to patient tolerance each week targeting 80% of the preoperatively determined MIP by the end of 8^th^ week after surgery. To ensure safety of the training, HR, blood pressure, oxygen saturation and respiratory rate will be monitored during training sessions.

Training will be performed and supervised by a single physiotherapist. This physiotherapist will be blinded to the group allocation and the study objectives. The training load was continuously monitored to ensure achievement of the target MIP. The patients will receive postoperative IMT for 20-30 min/session in the form of six sets, each set consisted of five deep breaths against the IMT device, with a short interval of 1-2minute rest between sets. Training will be performed on daily basis, twice per day till discharge from hospital. Training will be performed from comfortable sitting position using nose clip in every training session and patients were instructed to inspire through a mouth piece at the desirable training load.

On the day of discharge from hospital, all measurements will be repeated for all patients (pulmonary function measurements, maximal inspiratory pressure, and functional capacity). After discharge from the hospital, the patients will be instructed to visit the outpatient cardiopulmonary rehabilitation unit for continuing intervention program under supervision and for ensuring that patients will receive the proper training load. Training will be performed four times per week till the eighth postoperative week.

**Statistical analysis:**

Baseline descriptive statistics will be compared using independent t-tests for continuous data and Pearson chi- square analysis for categorical data.

Missing data will be remedied using last-observation-carried forward and all analyses will be based upon intention-to-treat analysis approach.

The Normality of data distribution will be analyzed with Shapiro Wilk test, indicating normal distribution of data (P˃0.05). Levin test will be used to verify data variance equality.

If the data are not normally distributed, Mann-Whitney U test will be used for between the group comparisons to establish the statistical significance among measurements. [Friedman test](https://www.sciencedirect.com/topics/medicine-and-dentistry/friedman-test) and [Wilcoxon Signed rank test](https://www.sciencedirect.com/topics/medicine-and-dentistry/wilcoxon-signed-ranks-test) will be used for within the group analysis to establish the statistical significance.

If the data are normally distributed, statistical analysis employed within subjects and between groups (groups X time) repeated measures Analysis of Variance (ANOVA) to investigate the effect of IMT on lung functions and functional capacity. Main effect of intervention; main effect of time as well as the interaction effect between intervention and time will be investigated. The repeated-measures data will be checked for sphericity violation using Mauchly’s test, the Greenhouse-Geisser correction was conducted when the sphericity was violated. Pairwise comparison and Post-hoc analyses with Bonferroni correction were conducted when there is significant group-time interaction.

Effect sizes for significant interaction effects will be reported as partial eta squared (η^2^) with the following classification to define small (η2 =0.01), medium (η2 =0.06), and, large (η2 =0.14) effect size[25].The data of peak amplitude, distal latency of dermatomal somatosensory evoked potential and pain intensity at two instances before stretching exercise session and at the end of the session. All data will be analyzed using SPSS version 20.0 software (SPSS Inc., Chicago, IL). The statistical significance level was set at (P˂0.05).

**Ethical consideration:**

Written informed consents will be obtained from participants before enrollment in the study. All participants will be informed about the study objectives before participation. Participants will be informed about their rights to refrain from the study at any time if they want.

References:

1. Kendall F, Oliveira J, Peleteiro B, Pinho P, Bastos PT. Inspiratory muscle training is effective to reduce postoperative pulmonary complications and length of hospital stay: a systematic review and meta-analysis. Disability and Rehabilitation. Taylor and Francis Ltd; 2018. pp. 864–882. doi:10.1080/09638288.2016.1277396

2. Galvan CCR, Cataneo AJM. Effect of respiratory muscle training on pulmonary function in preoperative preparation of tobacco smokers. Acta Cir Bras. 2007;22: 98–104. doi:10.1590/S0102-86502007000200004

3. Valkenet K, Van De Port IGL, Dronkers JJ, De Vries WR, Lindeman E, Backx FJG. The effects of preoperative exercise therapy on postoperative outcome: A systematic review. Clinical Rehabilitation. 2011. pp. 99–111. doi:10.1177/0269215510380830

4. Mans CM, Reeve JC, Elkins MR. Postoperative outcomes following preoperative inspiratory muscle training in patients undergoing cardiothoracic or upper abdominal surgery: A systematic review and meta analysis. Clin Rehabil. 2015;29: 426–438. doi:10.1177/0269215514545350

5. Snowdon D, Haines TP, Skinner EH. Preoperative intervention reduces postoperative pulmonary complications but not length of stay in cardiac surgical patients: A systematic review. J Physiother. 2014;60: 66–77. doi:10.1016/j.jphys.2014.04.002

6. Katsura M, Kuriyama A, Takeshima T, Fukuhara S, Furukawa TA. Preoperative inspiratory muscle training for postoperative pulmonary complications in adults undergoing cardiac and major abdominal surgery. Cochrane Database of Systematic Reviews. John Wiley and Sons Ltd; 2015. doi:10.1002/14651858.CD010356.pub2

7. Cordeiro ALL, de Melo TA, Neves D, Luna J, Esquivel MS, Guimarães ARF, et al. Inspiratory muscle training and functional capacity in patients undergoing cardiac surgery. Brazilian Journal of Cardiovascular Surgery. 2016. pp. 140–144. doi:10.5935/1678-9741.20160035

8. Brocki BC, Andreasen JJ, Langer D, Souza DSR, Westerdahl E. Postoperative inspiratory muscle training in addition to breathing exercises and early mobilization improves oxygenation in high-risk patients after lung cancer surgery: A randomized controlled trial. Eur J Cardio-thoracic Surg. 2016;49: 1483–1491. doi:10.1093/ejcts/ezv359

9. George R , Light R and MM. Essentials of pulmonary and critical care medicine, Chest Medicine. 4th ed. Essentials of pulmonary and critical care medicine, Chest Medicine. 4th ed. New York and London; 2000. pp. 192–193.

10. Santos BFA, Souza HCD, Miranda APB, Cipriano FG, Gastaldi AC. Performance in the 6-minute walk test and postoperative pulmonary complications in pulmonary surgery: An observational study. Brazilian J Phys Ther. 2016;20: 66–72. doi:10.1590/bjpt-rbf.2014.0119

11. Keeratichananont W, Thanadetsuntorn C, Keeratichananont S. Value of preoperative 6-minute walk test for predicting postoperative pulmonary complications. Ther Adv Respir Dis. 2016;10: 18–25. doi:10.1177/1753465815615509

12. Westerdahl E, Jonsson M, Emtner M. Pulmonary function and health-related quality of life 1-year follow up after cardiac surgery. J Cardiothorac Surg. 2016;11: 99. doi:10.1186/s13019-016-0491-2

13. Gomes Neto M, Martinez BP, Reis HFC, Carvalho VO. Pre- and postoperative inspiratory muscle training in patients undergoing cardiac surgery: Systematic review and meta-analysis. Clinical Rehabilitation. 2017. pp. 454–464. doi:10.1177/0269215516648754

14. Karanfil EOT, Møller AM. Preoperative inspiratory muscle training prevents pulmonary complications after cardiac surgery – a systematic review. Danish Medical Journal. Danish Medical Association; 2018.

15. Chandra A, Srivastava S, Dilip D. Spirometric Changes following Open-Heart Surgery on Rheumatic Mitral Valves. Asian Cardiovasc Thorac Ann. 1998;6: 28–33. doi:10.1177/021849239800600106

16. Luthra S, Dhaliwal R, Rana S, Behera D, Saxena P. Early changes in pulmonary functions after mitral valve replacement. Ann Thorac Med. 2007;2: 111. doi:10.4103/1817-1737.33699

17. Hassan AM, El Nahas NG. Efficacy of cardiac rehabilitation after percutaneous coronary intervention. Int J PharmTech Res. 2016;9: 134–141. Available: https://scholar.cu.edu.eg/sites/default/files/nessrien_elnahass/files/134-141v9n4pt-aly-_nesreen.pdf

18. Miskovic A, Lumb AB. Postoperative pulmonary complications. BJA Br J Anaesth. 2017;118: 317–334. doi:10.1093/bja/aex002

19. Chou C-H, Chen C-Y. Inspiratory muscle training for reducing postoperative pulmonary complications: A simple method that we hope is effective EDITORIAL COMMENTARY THOR. J Thorac Cardiovasc Surg c. 2018;156: 1301. doi:10.1016/j.jtcvs.2018.03.031

20. Luiz Lisboa Cordeiro A, Araújo de Melo T, Neves D, Luna J, Souza Esquivel M, Raimundo França Guimarães A, et al. Inspiratory Muscle Training and Functional Capacity in Patients Undergoing Cardiac Surgery. Brazilian J Cardiovasc Surg Braz J Cardiovasc Surg. 2016;31: 140–144. doi:10.5935/1678-9741.20160035

21. Shakouri SK, Salekzamani Y, Taghizadieh A, Sabbagh-Jadid H, Soleymani J, Sahebi L, et al. Effect of respiratory rehabilitation before open cardiac surgery on respiratory function: a randomized clinical trial. J Cardiovasc Thorac Res. 2015;7: 13–7. doi:10.15171/jcvtr.2015.03

22. Borghi-Silva A, Mendes RG, Costa F de SM, Di Lorenzo VAP, Oliveira CR de, Luzzi S. The influences of positive end expiratory pressure (PEEP) associated with physiotherapy intervention in phase I cardiac rehabilitation. Clinics (Sao Paulo). 2005;60: 465–472. doi:10.1590/S1807-59322005000600007

23. Kamel SMM, Obaya HE. Effect of cardiac rehabilitation on ejection fraction after percutaneouscoronary intervention. Int J PharmTech Res. 2016;9: 127–133.

24. Sasaki M, Kurosawa H, Kohzuki M. Effects of inspiratory and expiratory muscle training in normal subjects. J Japanese Phys Ther Assoc. 2005;8: 29–37. doi:10.1298/jjpta.8.29

25. Lakens D. Calculating and reporting effect sizes to facilitate cumulative science: A practical primer for t-tests and ANOVAs. Front Psychol. 2013;4: 863. doi:10.3389/fpsyg.2013.00863
